# Supplementary figures and images for: GeneTonic: an R/Bioconductor package for streamlining the interpretation of RNA-seq data
Source: BMC Bioinformatics. 2021 Dec 23;22:610. doi: 10.1186/s12859-021-04461-5 (PMC8697502; doi:10.1186/s12859-021-04461-5)

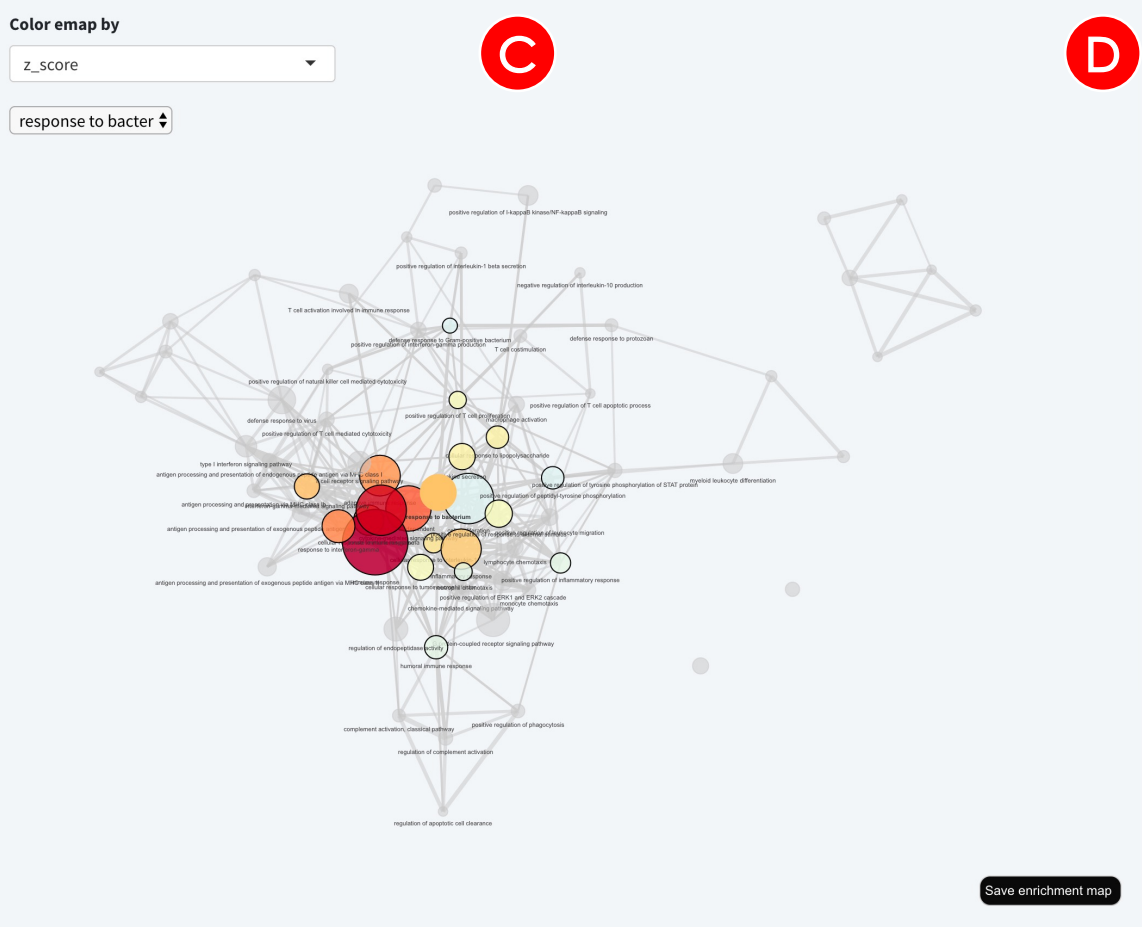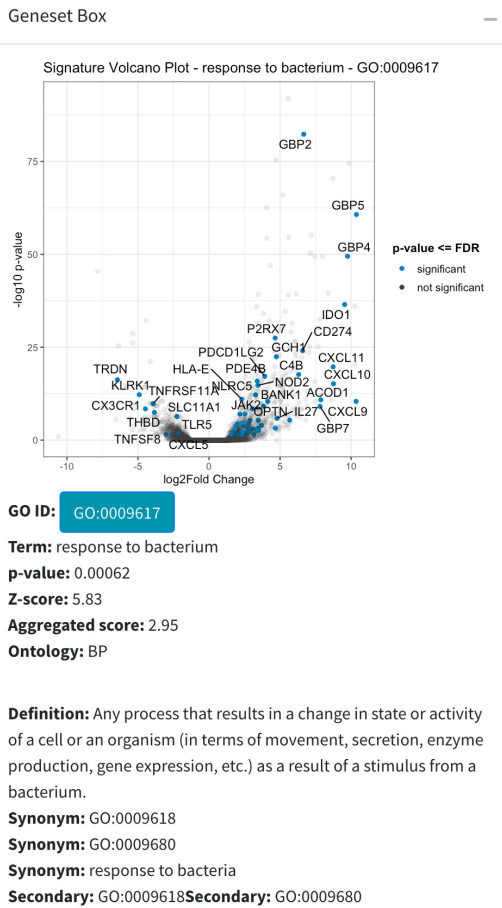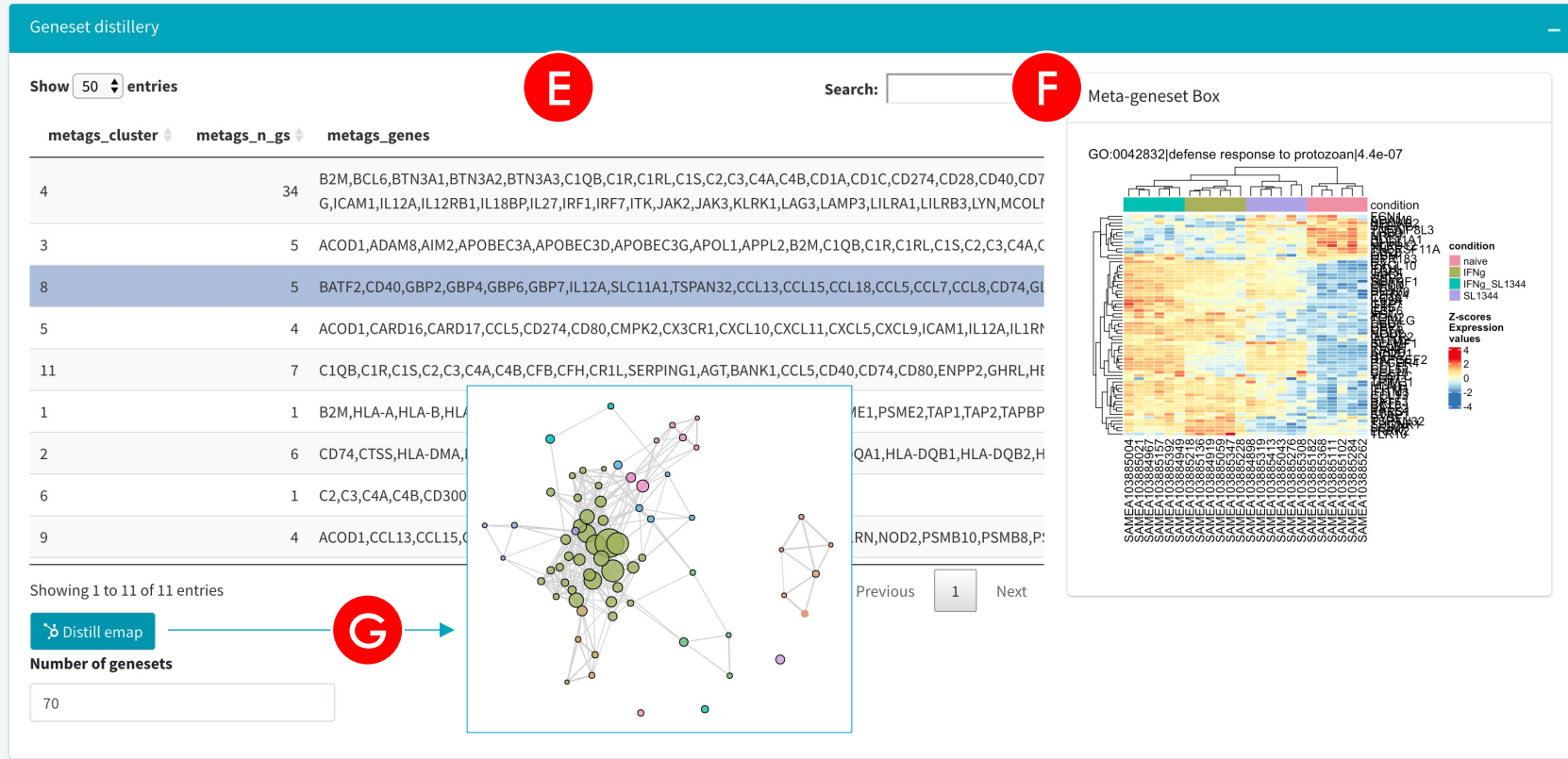

Supplement: Supplementary file 4 — Additional file 4: Fig. S1. Screenshot of the Enrichment Map panel in the GeneTonic application. The sidebar menu (A) controls the main navigation in the app, and a common set of options is toggled with the cogs icon (B). The main area of the Enrichment Map panel (C) contains an interactive graph for the enrichment map of the genesets, connected according to their similarity, and color coded according to the specified geneset property (here, the Z-score). Upon clicking on any geneset, a Geneset Box (D) is displayed for further exploration (e.g. to show a volcano plot with the geneset members labelled). The geneset distillery (E) enables the exploration of meta-genesets, derived by computing clusters on the graph object underlying the enrichment map. From the tabular representation, it is possible to visualize meta-genesets as heatmaps (F), or display a modal popup containing the enrichment map where the cluster assignments of the genesets are shown (G). [file 12859_2021_4461_MOESM4_ESM.pdf]
